# Clinicopathologic Study of Oral Mucosa-Derived Hamartomas and Choristomas and Literature Review

**DOI:** 10.1155/ijod/5750421

**Published:** 2025-11-10

**Authors:** Ianca Daniele Oliveira de Jesus, Anaíra Ribeiro Guedes Fonseca Costa, Débora de Oliveira Santos, Sérgio Vitorino Cardoso, Nayara Rúbio Diniz Del Nero, Adriano Mota Loyola, Paulo Rogério de Faria

**Affiliations:** ^1^Department of Oral Pathology, School of Dentistry, Federal University of Uberlândia, Uberlândia, Brazil; ^2^Department of Immunology, Institute of Biomedical Science, Federal University of Uberlândia, Uberlândia, Brazil

**Keywords:** choristoma, epidemiologic studies, hamartoma, mouth mucosa, soft tissue injuries

## Abstract

This study aimed to investigate the sociodemographic and clinicopathological data of oral soft tissue hamartomas and choristomas. A cross-sectional study was delineated to quantitatively analyze oral soft tissue hamartomas and choristomas cases diagnosed between 1978 and 2025 in a Brazilian Oral Pathology Service. The patient's file raised data regarding the clinical parameters, demographic data, diagnostic imaging, and histopathological features. A review of case reports published in the literature was also performed, analyzed, and compared to our casuistic. Fifteen cases were retrieved from our archive: 60.00% hamartomas and 40.00% choristomas. Females were more affected than males (53.33% vs. 46.67%). The ages of the affected patients varied from newborns to older people. Clinically, most of the lesions were nodular, had an oral mucosa-like color, and were painless. The buccal gingiva was the most prevalent region affected by the lesions (53.33%). All cases were treated by surgery. Although uncommon, oral soft tissue hamartomas and choristomas mainly affect people of different ages, from newborns to older people. They should be diagnosed promptly to offer better therapeutic management, avoiding invasive treatments that contradict their benign nature.

## 1. Introduction

Hamartoma is an excessive proliferation of disorganized, normal tissue indigenous to the injury site, while choristoma is defined as a proliferation of well-organized, normal tissue in an abnormal location [1]. Hamartomas and choristomas of the oral cavity are uncommon lesions [2, 3]. Both lesions can present various clinical and histopathological aspects [4]. Oral hamartomas are comprised mainly of odontogenic and non-odontogenic epithelial tissues, but muscle, vessels, nerves, fat, and salivary gland tissues can also be found [5–8]. Oral choristomas comprise cartilage, bone, thyroid, and gastric tissue [9]. They mainly affect young people, but variations in the frequency of these lesions between patients' genres have been reported in the literature [1, 10]. The most typical sites of occurrence in the oral cavity are the tongue, gingiva, cheek, lip, floor of the mouth, oral vestibule, salivary gland, palate, and jaws [11–16]. Surgical excision is the treatment of choice, and the recurrence rate is relatively rare [17].

Casuistic studies exclusively describing the demographic and clinicopathologic features of oral soft tissue hamartomas and choristomas have yet to be reported in the literature. So, this study purposes to report the demographic and clinicopathological characteristics of oral soft tissue hamartomas and choristomas retrieved from a Brazilian regional reference laboratory of oral pathology. Furthermore, this study also includes a comprehensive review of the literature regarding published case reports depicting the lesions' clinical, microscopic, demographic, radiographic, and imagiological aspects.

## 2. Material and Methods

### 2.1. Case Selection

Oral soft tissue hamartoma and choristoma cases were retrospectively collected from the files of the Department of Oral Pathology, School of Dentistry, Federal University of Uberlândia, Brazil, between 1978 and 2025, after approval from the Institutional Ethics Committee on Human Research (30671120.3.0000.5152). Data collection was performed following the Declaration of Helsinki. The need for informed consent was waived by the Ethics Committee considering the retrospective nature of this observational study and the removal of personally identifiable information from the dataset. Hematoxylin and eosin-stained slides from all selected cases were raised and used by an experienced oral pathologist to review the diagnoses on light microscopy. Data concerning age, gender, evolution, location, symptoms, clinical aspects, size, histopathological diagnosis, and treatment were gathered from the patient's record.

### 2.2. Literature Review

A comprehensive literature review was performed using electronic databases (Lilacs, Scielo, Pubmed, Web of Science, and Scopus) to identify only oral soft tissue hamartomas and choristomas case reports and case series. The papers investigated were those published from 1956 to May 2025. The following search terms were used: “hamartomas” OR “choristomas” AND “oral mucosa” OR “oral soft tissue.” Additionally, a manual search was conducted by cross-referencing the retrieved manuscripts. The following inclusion criteria were used to select the publications: well-documented case reports or case series (clinical and histopathologic images) regarding patients diagnosed with soft tissue hamartomas and choristomas fully available online in scientific databases or printed versions. The exclusion criteria were case reports and case series with no or suboptimal histological documentation confirming the diagnosis, literature reviews, surveys, multiple hamartomas associated with syndromes, sebaceous glands, melanocytic lesions, and odontomas of the jaws, the most common hamartomas of the oral cavity. From this extensive review of the literature, 540 articles were retrieved. After a thorough analysis, 291 articles with a sample size of 337 lesions were included in this study. Clinical parameters, radiographic imaging, and histopathological features were collected from the studies and tabulated in an Excel Microsoft spreadsheet. Descriptive statistics (mean) were applied to characterize our sample. Fisher's exact test was used to identify associations between gender and age with hamartomas and choristomas. *p*  < 0.05 was considered statistically significant.

## 3. Results

### 3.1. Clinical Data

The overall clinicopathological data of our cases are depicted in Table 1. The patient sample consisted of eight women and seven men, yielding a 1.14:1 female-to-male ratio. Half of the patients were diagnosed between the first and fourth decade of life. The age range widely varied, with a mean of 34.2 years. The mean age was 18.2 for males and 30.9 for females. Interestingly, most of them (10 cases, 66.66%) were diagnosed in the adult population (>18 years old). The buccal gingiva (eight cases, 53.33%) was the most prevalent region affected by the lesions, followed by the tongue, labial mucosa, and palate (two cases each, 13.33%). Asymptomatic lesions predominated (nine cases, 60%). About 46.66% of the lesions were non-congenital, and 26.66% presented an evolution time of up to 1 year.

Clinically, the lesions presented as pediculated nodules (four cases, 26.66%), sessile nodules (3 cases, 19.98%), and plaque/papule (one case, 6.66%). The vast majority were solitary lesions (14 cases, 93.33%). The size ranged from 0.5 to 8 cm in their largest dimension, with a mean size of 1.75 cm. The lesions' consistency varied from soft to hard. Lesions clinically characterized as mobile and fixed were seen in four cases (26.66%) and two cases (13.33%), respectively. Regarding the color features, pink was the most frequent (10 cases, 66.66%), followed by red (three cases, 20%) and white (two cases, 13.33%). About initial clinical hypothesis, fibrous hyperplasia prevailed (four cases, 26.66%), which was followed by congenital epulis, sialolithiasis, peripheral ossifying fibroma, bone tissue, papilloma, and dental follicle (one case each, 6.66%). Five out of 15 (33.33%) did not have any information clinical diagnosis in the patient's record.

About initial clinical hypothesis, fibrous hyperplasia prevailed (four cases, 26.66%), which was followed by congenital epulis, sialolithiasis, peripheral ossifying fibroma, bone tissue, papilloma, and dental follicle (one case each, 6.66%). Five out of 15 (33.33%) did not have any information concerning clinical diagnosis in the patient's record.

### 3.2. Histopathological Diagnosis

**Figure 1:**
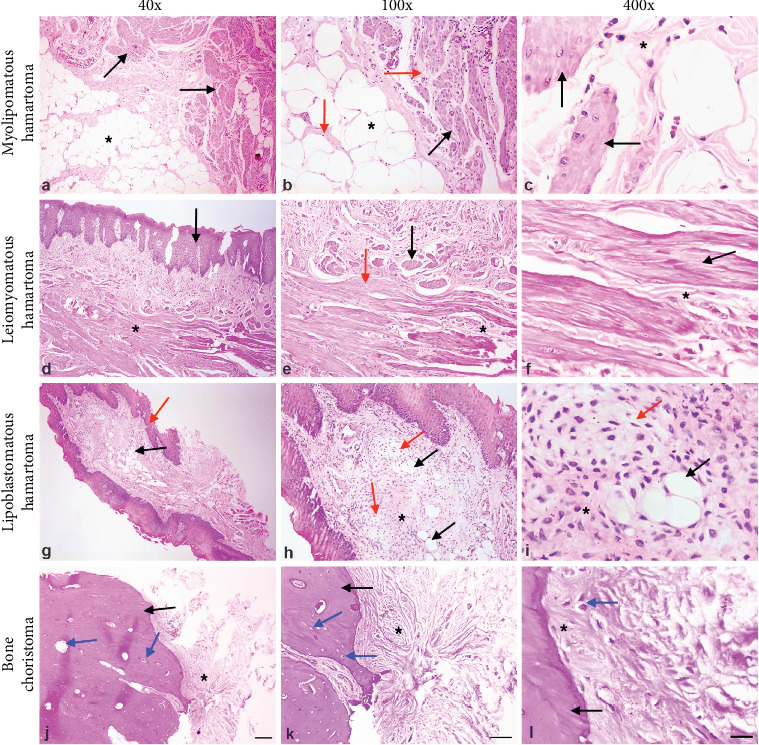
Histopathological aspects of oral soft tissue-derived hamartomas and choristoma cases stained with hematoxylin and eosin from our casuistic. (a) Tissue section showing a cluster of mature adipocyte cells (asterisk) and fascicles of well-differentiated smooth muscle cells (black arrows). (b) Note some adipocyte (asterisk) and smooth muscle bundles (black arrow) encircled by collagenous fibers (red arrows). (c) See small bundles of smooth muscle cells with pink-staining cytoplasm and cigar-shaped nuclei (black arrows) surrounded by thin collagenous fibers (asterisk). (d) Oral mucosa covered by parakeratotic squamous stratified epithelium exhibiting acanthosis and hyperplasia (black arrow) and presenting in the reticular lamina of the connective tissue several fascicles composed of well-differentiated smooth muscle cells (asterisk). (e) Note in the reticular lamina of the connective tissue several longitudinal- and cross-sectioned bundles of smooth muscle fascicles (black and red arrows) surrounded by a loose connective tissue (asterisk). (f) Longitudinally sectioned fascicles of smooth muscle cells (black arrow) intermixed with thin collagenous fibers and fibroblasts (asterisk). (g) Oral mucosa lined by parakeratotic squamous stratified epithelium (red arrow) presenting in the connective tissue some mature fat cells (black arrow). (h) Connective tissue presenting a cluster of clear (red arrows), acidophilic (asterisk), and mature adipocyte cells (black arrows) (i). In more detail, clear cells (red arrow) are characterized by multivacuolated cytoplasm and ovoid nuclei intermixed with a few groups of univacuolar signet-ring mature adipocyte (black arrow) and acidophilic cells (asterisk). (j) Tissue section revealing a lamellar bone tissue (black arrow) presenting some haversian systems (osteons; blue arrows) and dense connective tissue adhered to it (asterisk). (k) Mature bone tissue (black arrow) with some osteocyte-containing lacunae (blue arrows) surrounded by dense connective tissue (asterisk). (l) In more detail, mature bone trabeculae (black arrow) presenting at the periphery a deposition of nonlamellar bone (asterisk) with some rounded osteocytes (blue arrow) embedded in the osteoid matrix (40x scale bar: 100 um; 100x scale bar: 50 um; 400x scale bar: 25 μm).

**Figure 2:**
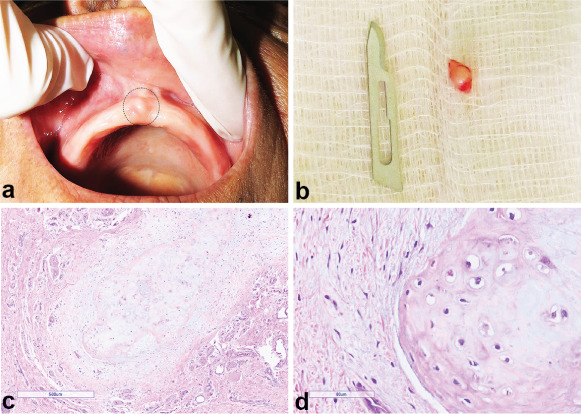
Choristoma chondroid case from our casuistic. (a) Clinical image showing a nodular lesion localized at the alveolar ridge. (b) Lesion removed after excisional biopsy. (c) Histopathological aspect of the lesion revealing a mature hyaline cartilage tissue surrounded by a fibromyxoid tissue (H&E; original magnification 100x). (d) Details of the hyaline cartilage showing chondrocytes inside their lacunae displaying round nuclei and homogeneous cytoplasmic basophilia and surrounded by cartilaginous matrix secreted by them (H&E; original magnification 400x).

Figure 1 depicts some histopathological aspects of hamartomas and choristomas cases from our casuistic. Among the 15 patients, nine cases were diagnosed as hamartomas (60.00%) and six as choristomas (40.00%). The mean age for hamartoma- and choristoma-affected patients was 34 years old (range: 0.3–60 years old) and 46 years old (range: 25–68 years old), respectively. However, there was no significant association between hamartomas and choristomas with age and gender (Tables 2 and 3). Regarding hamartoma cases, the following diagnoses were obtained: leiomyomatous hamartoma and odontogenic hamartoma with two cases each (13.33%); and nonspecific hamartoma, myolipomatous hamartoma, lipoblastomatous hamartoma, odontogenic epithelial hamartoma, and giant cell hamartoma with one case each (6.66%). The primary tissue types detected in the hamartoma lesions were smooth muscle and odontogenic tissues. The histopathological diagnoses for choristoma cases were chondroid (one case, 6.66%) and osseous (five cases, 13.33%).  Figure 2 shows clinical, macroscopy, and histological aspects of the choristoma chondroid from our casuistic.

### 3.3. Imaging/Radiographic Diagnosis

Radiographic and imagiological exams were not commonly used to diagnose such lesions in our case, with the periapical and panoramic radiographs employed in two cases (13.33%).

### 3.4. Treatment and Follow-up

All lesions were removed by surgery under local anesthesia and referred to the oral pathology laboratory for histopathological diagnosis. No clinical intercurrences were observed during or after surgery, but three cases recurred: two bone choristomas and one smooth muscle hamartoma. No information on the follow-up could be obtained from the dental records of the affected patients.

### 3.5. Literature Review

The demographic and clinicopathological data of case reports published in the literature are detailed in Tables 4 and 5.

About 89.32% and 10.68% of the lesions were non-congenital and congenital, respectively. The patient sample consisted of 178 women and 152 men. The newborns (28.78%) were the most affected, followed by adults (22.85%) and children (21.36%). Regarding the lesion's location, the tongue (192 cases, 56.97%), more precisely the dorsum, posterior dorsum, and base, was the most compromised region, followed by the gingiva (44 cases, 13.06%), palate (26 cases each, 7.72%), and floor of the mouth (24 cases, 7.12%). There was a predominance of asymptomatic lesions (99 cases, 29.38%). The commonly reported symptoms were dysphagia (20 cases, 5.93%) and dyspnea (15 cases, 4.45%). The duration of the lesions ranged from 1 month to 5 years (60 cases, 17.80%). Clinically, the surface texture was primarily smooth (15.13%) and lobulated (5.04%); consistency ranged from soft to hard. Regarding the color features, pink was the most frequent (71 cases, 21.07%), followed by white (15 cases, 4.45%) and red (eight cases, 2.37%).

Among the 337 cases gathered from the literature, 117 were diagnosed as hamartomas (34.72%) and 220 as choristomas (65.28%) (Tables 6 and 7). For hamartomas, nonspecific ones (32 cases, 27.35%), leiomyomatous (29 cases, 24.79%), and peripheral compound odontomas (eight cases, 6.84%) were the most frequent diagnoses (Table 6). Regarding choristomas, cystic (54 cases, 24.55%), osseous (40 cases, 18.18%), neural (28 cases, 12.73%), and thyroid ones cartilaginous (24 cases, 10.91%) prevailed as the main diagnoses (Table 7).

Radiographic and imagiological exams were not usually used for diagnostic purposes. However, in some studies, computed tomography scan (15.43%), magnetic resonance imaging (8.60%), and intraoral (periapical and occlusal, 2.64%) and extraoral (panoramic, 5.04%) radiographs were reported. Ultrasonography was mainly used for prenatal diagnosis (3,26%).

All lesions were removed entirely by surgery under local anesthesia and submitted to histopathological examination. No case of recurrence was reported in the literature.

## 4. Discussion

In this work, we reported the demographic and clinicopathological features of hamartomas and choristomas that developed in the oral mucosa, excluding from this analysis all jawbone lesions and a complete review of the pertinent literature. Our results are closely related to the literature, except for the predominance of hamartomas over choristomas, the location of the lesions, and the age of the affected patients.

Hamartoma represents an excessive proliferation of disorganized tissue indigenous to a particular region, whereas choristoma is defined as a well-organized mass composed of normal, nonindigenous tissue [18]. Even though the pathogenesis of both lesions is unknown [17], the abnormal cranial neural crest (CNC) developmental pathway has been related to their development since it is the primary source of craniofacial ectomesenchyme and mesenchymal stem cells that, under uncharacterized stimuli, could give rise to both lesions [3]. Another hypothesis is about lingual bone choristoma development, in which the CNC pluripotent cells' differentiation-inducing traumatic/chronic inflammation has been postulated [19]. However, further studies are necessary to figure out their pathogenesis.

Previous oral hamartomas and choristomas reports have shown that both lesions mainly affect young female patients [1]. Indeed, the literature review revealed a slight prevalence for females (52.82%) and newborns/children (50.14%), although 33.53% were diagnosed in adults. Comparatively, our casuistic also showed a slight preference for females, but surprisingly, most of the lesions were diagnosed in the adult population. This difference may be explained by either a reduced sample size of our sample or that this represents the real epidemiology of hamartomas and choristomas affecting the oral soft tissues. Despite that, these data corroborate that both lesions are preferentially detected in young female patients. However, they may also occur in adults, especially when these lesions develop in the soft tissue of oral mucosa.

Another Brazilian study identified a frequency of 1.83% and 0.01% of hamartomatous and choristomatous lesions, respectively, in a 16,412 patient cohort, reporting similar findings [20]. According to the authors, 50.2% of hamartomas occurred in adults and 60.7% in females, with a female-to-male ratio of 1.5:1. It is important to note that they included hard tissue lesions that were not approached in our study, such as odontoma, exostoses, and adenomatoid odontogenic tumor, as well as other soft tissue alterations, that is, vascular malformation, nevus, lymphatic malformations, and hemangioma. Despite the reportedly large sample size (*n* = 181, 60.7%), most comprised of odontomas (*n* = 114), vascular malformations (*n* = 47), and nevus (*n* = 44).

We decided to include only soft tissue hamartomas because odontomas are already a well-described entity, so we could explore other rare diseases. Although once considered an hamartomatous odontogenic lesion, the adenomatoid odontogenic tumor is currently classified as a benign neoplasm by the latest WHO Classification of Head and Neck tumors [21]. Similarly, recent studies have shown torus and exostoses to result from the interplay between genetic and environmental factors, the latter mainly dental wear and tooth loss, rather than having a developmental etiology [22, 23]. Other oral soft tissue alterations, such as nevi, lymphovascular malformations, and hemangiomas, were also excluded because they don't always meet certain hallmarks for hamartomas [17]. Melanocytic nevi, for instance, are frequently referred as benign neoplasms [24]. Concerning vascular lesions, the literature is elusive on the distinction between hamartomas and benign neoplasms [25]; the ISSVA currently classifies vascular anomalies under vascular tumors or vascular malformations [26]. The current WHO Classification of Head and Neck tumors, in turn, describes hemangiomas as benign vascular neoplasms and lymphangiomas as benign vascular lesions [27]. In this context, our study provides valuable clinicopathological data on rare mucosal entities with confirmed hamartomatous nature that are little described in the literature. Moreover, it is out of scope of this work to discuss the differences between hamartomatous lesions and neoplasia.

Most oral soft tissue hamartomas and choristomas often arise in the anterior two-thirds of the tongue, which may be explained by the proximity of the endoderm precursor derivatives to the branchial arches during tongue development [28, 29]. Our revision confirmed this finding, with 192 cases (56.97%) originating at this anatomical site (44 and 128 cases of hamartomas and choristomas, respectively). In addition, other oral regions may also be compromised, including the gingiva, the second most common site observed in the literature review (44 cases, 13.06%). Conversely, the gingiva was the most affected region (53.33%) in our casuistic, followed by the tongue, and this difference could be closely related to our retrospectively convenient sample size. Together, these data uncover that the tongue and the gingiva are critical sites for oral soft tissue hamartomas and choristomas development.

Clinically, oral soft tissue hamartomas and choristomas are slowly progressive, self-limited solitary or multiple tumor-like lesions [30, 31]. The literature review showed that 82.20% were nodular, 22.74% pedunculated, and 96.14% solitary, and the size ranged from 1 to 7 cm in dimension. In our casuistic, 66.66% and 93.33% of the hamartomas and choristomas were nodular and solitary lesions, respectively, with a mean size of 2.20 cm, ranging from 0.5 to 8 cm, which reflects their slow growth rate. Additionally, in the literature review, pink (71 cases, 21.07%), white (15 cases, 4.45%), and red (eight cases, 2.37%) were the most common color features, as observed in our casuistic. Altogether, nodular, pink, and soft oral mucosal lesions should be included in the differential diagnosis of hamartomas and choristomas.

As for symptoms, both lesions are usually asymptomatic and rarely cause serious complications, so they are usually detected incidentally in the clinic [10]. In the literature review, the lesions were typically asymptomatic, but dysphagia, dysphonia, dyspnea, and emesis were reported in some studies [11]. It is ascertained that affected patients hardly identify painless lesions except when they harbor lesions with perceptible growth accompanied by or without symptoms [32]. In our casuistic, pain was reported only in three cases at presentation; the remaining ones were asymptomatic, as observed in the literature.

The histopathological examination of these lesions is essential for excluding other lesions that show overlapping clinical and radiological features in the pediatric population, including benign and malignant ones [17, 29]. In the literature review, choristomas (220 cases, 65.28%) were more prevalent than hamartoma (117 cases, 34.72%), whereas, in our casuistic, hamartomas (60.00%) outnumbered choristomas (40.00%). Regarding the histopathological diagnosis of hamartomas, the most frequent ones were leiomyomatous, followed by peripheral compound odontoma, although 32 cases were hamartomas without a specific diagnosis. Most choristoma lesions reported in the literature were cystic, osseous, neuronal, and cartilaginous. Similarly, leiomyomatous and odontogenic hamartomas predominated in our sample, whereas cartilaginous and osseous tissues prevailed in choristoma lesions. These findings reinforce the importance of microscopic analysis in establishing the correct diagnosis.

Radiographic and imagiological exams may be used for diagnosing and surgical planning since they help determine the lesion's nature, extension, location, and relationship with critical anatomical structures, especially the airway [1, 33, 34]. In our sample, two cases (13.33%) reported using periapical and panoramic radiographs during the clinical exam compared to 102 cases (33.77%) in the revised literature. Interestingly, 46 cases (15.23%) from the literature review mentioned using CT scans (46 cases, 15.23%) for diagnostic purposes, but none in our casuistic. Thus, from these findings, it is possible to conclude that, for the most part, the clinical features of hamartomas and choristomas mostly outweigh the use of complementary exams for their diagnosis. However, this does not apply to intraosseous, peripheral cystic, congenital, and large lesions where periapical and panoramic radiographs, CT scans, MRI, and ultrasonography may be well indicated.

The differential diagnosis may include congenital mucocele or ranula, lymphatic or vascular malformation, dermoid and odontogenic cysts, congenital granular cell tumor, teratoma, reactive lesions, and salivary gland tumor [35, 36]. One lesion that deserves some comment is teratomas, rare oral cavity lesions composed of the three primordial germ layers, that is, the ectoderm, the mesoderm, and the endoderm [37]. They affect mainly the tongue and can contain skin, hair, mucous membranes, and cartilage tissues. As they are large lesions, oral teratomas can cause airway obstruction in children and infants [38]. Although there is a remarkable similarity between hamartomas, choristomas, and teratomas, differential diagnosis is made based on their potential growth and histopathological features. Therefore, hamartomas and choristomas of oral soft tissue origin should not be overlooked. Moreover, all excised lesions must be sent for histopathological analysis to confirm the diagnosis and exclude other lesions, especially those with more aggressive behavior.

The treatment of both lesions is the conservative surgical excision, with an excellent prognosis due to minimal chances of recurrence [39]. Indeed, in all cases reported in the literature, surgical excision was the treatment of choice, with no signs of recurrence, showing the efficiency of this approach. The same treatment was adopted in our study, but three recurrence cases were found. It is well known that oral hamartomas and choristomas are not expected to recur when proper treatment is employed. However, oral cyst choristoma may result in recurrence when aspiration and marsupialization are the treatment of choice [34]. Even though we cannot explain the recurrence cases in our casuistic, it is worth emphasizing that they might unexpectedly occur. Thus, a follow-up longer than 1 year should be necessary for some cases.

Despite the extensive literature review, our investigation has some limitations, which should be acknowledged. First, our study was based on a retrospective and single-center design, reflecting a regional diagnostic practice or referral patterns that could not be generalized to other populations. Second, despite a long data collection period employed in our study, we obtained a relatively small sample size, limiting our ability to draw robust conclusions about clinicopathologic patterns or demographic trends of both lesions. Nevertheless, this is an essential investigation for clinicians because it allows them to be aware of such lesions and their clinical, radiographic, and histopathological characteristics.

## 5. Conclusion

In conclusion, oral soft tissue hamartomas and choristomas are uncommon compared to odontomas and may be present in children, adults, and older people. Clinical diagnosis coupled with microscopic analysis is essential to make the correct diagnosis. Although these lesions are developmental changes, they can cause morbidity by obstructing or compressing anatomical structures adjacent to their growth sites. While this study provides valuable and detailed epidemiological data on soft tissue hamartomas and choristomas diagnosed at a single institution—excluding odontomas (i.e., hard tissue hamartomas)—its findings must be interpreted cautiously due to inherent limitations. The relatively small number of cases, combined with the retrospective and single-center nature of the study, may limit the generalizability of the results. Nevertheless, by integrating our institutional data with a literature review, this work offers a meaningful contribution to understanding these rare entities, which may assist both general and specialist practitioners in clinical practice.

## Figures and Tables

**Table 1:** Distribution of oral soft tissue hamartoma and choristoma cases according to sociodemographic and clinicopathological features.

Patient	Age	Gender	Evolution	Location	Symptoms	Clinical appearance	Size	Histopathological diagnosis
1	28 y	F	NI	Dorsum of tongue	Absent	Nodule, normal color, pedunculated, mobile	3 cm	Hamartoma
2	25 y	F	NI	Superior labial mucosa	NI	Nodule, white color, hard consistency	NI	Osseous choristomas
3	60 y	F	Congenital	Inferior lip, anterior region	Absent	Tumor, normal color, fibrous consistency, pedunculated, mobile	8 cm	Myolipomatous hamartoma
4	16 y	F	NI	Gingiva between the canine and first premolar	Absent	Tumor, erythematous color, hard consistency, deep insertion, fixed	1.5 cm	Odontogenic hamartoma
5	65 y	M	5 y	Sublingual region	Present	White color, hard consistency, deep insertion	0.5 cm	Osseous choristomas
6	10 y	M	1 y	Gingiva, region of tooth 46	Absent	Nodule, normal color	1 cm	Odontogenic hamartoma
7	37 y	F	6 mo	Gingiva, upper premolar region	Absent	Nodule, normal color, hard consistency	0.5 cm	Osseous choristomas
8	8 mo	M	Congenital	Mucosa of the upper alveolar ridge	Absent	Nodule, normal color, borrachoid consistency, pedunculated, mobile	1 cm	Leiomyomatous hamartoma
9	52 y	F	1 y	Gingiva, upper midline region	Absent	Nodule, normal color	0.5 cm	Chondroid chorystoma
10	28 y	M	2 y	Palate mucosa	Absent	Plaque/papule, red in color, warty consistency	1 cm	Smooth muscle hamartoma
11	4 mo	F	Congenital	Dorsum of tongue	Present	Nodule, normal color	1 cm	Lipoblastomatous hamartoma
12	29 y	F	1 y	Mucosa of the retromolar trigone	Present	Nodule, normal color, hard consistency, sessile	2 cm	Osseous choristomas
13	19 y	M	NI	Mucosa adhered to the lower third molar	NI	Red color, soft consistency, adhered to the tooth, fixed	NI	Odontogenic epithelial hamartoma
14	7 y	M	NI	Gingiva, region of tooth 46	NI	Normal color	NI	Giant cell odontogenic hamartoma
15	68 y	M	3 y	Mucosa of hard to soft palate	Absent	Ovoid nodule, pinkish color, soft consistency, pedunculated, mobile	2 cm	Osseous choristomas

Abbreviations: F, female; M, male; mo, months; NI, not informed; y, years.

**Table 2:** Association between gender and hamartoma and choristoma cases gathered from our casuistic.

Gender	Hamartoma	Choristoma	Total	*p* value
Male	5	2	7	0.61
Female	4	4	8	
Total	9	6	15	—

*Note:* Fisher's exact test, *p*  < 0.05.

**Table 3:** Association between age and hamartoma choristoma cases gathered from our casuistic.

Age	Hamartoma	Choristoma	Total	*p* value
<30 yo	6	2	8	0.31
>30 yo	3	4	7	
<50 yo	8	3	11	0.23
>50 yo	1	3	4	

*Note:* Fisher's exact test, *p*  < 0.05.

Abbreivation: yo, years old.

**Table 4:** Demographic distribution hamartomas and choristomas reported in the literature.

Variables	Number of cases	%
Age		
RN (up to 1 year)	97	28.78
1–10 years	72	21.36
10–18 years	31	9.20
>18−50 years	77	22.85
>50 years	36	10.68
NI	24	7.12
Sex		
Female	178	52.82
Male	152	45.10
SI	7	2.08
Appearance		
Congenital	36	10.68
Noncongenital	301	89.32
Evolution time		
Up to 1 year	71	21.07
>1 year	32	9.50
SI	234	69.44
Total	337	100

*Note*: RN, newborn.

Abbreviation: NI, not informed.

**Table 5:** Clinicopathological distribution hamartomas and choristomas reported in the literature.

Variables	Number of cases	%
Type of lesion		
Nodule	277	82.20
Sessil	21	7.58
Pediculated	63	22.74
Bubble	53	15.73
Other	3	0.89
NI	4	1.19
Symptoms		
Present	15	4.45
Absent	99	29.38
NI	223	66.17
Number of lesions		
Solitary	324	96.14
Multiple	13	3.86
Mobility		
Present	35	10.39
Absent	41	12.17
NI	261	77.45
Color		
Pink	71	21.07
Red	8	2.37
White	15	4.45
Other	24	7.12
NI	219	64.99
Consistency		
Soft/smooth	40	11.87
Hard	35	10.39
Fibrous	60	17.80
Other	6	1.78
NI	196	58.16
Size		
Up to 1 cm	102	30.27
>1–3 cm	103	30.56
>3 cm	24	7.12
NI	108	32.05
Surface		
Smooth	51	15.13
Lobulated	17	5.04
Other	13	3.86
NI	256	75.96
Total	337	100

Abbreviation: NI, Not informed.

**Table 6:** Histopathological diagnosis of hamartomas reported in the literature.

Types of hamartomas	Number of cases	%
Hamartomatous angiolipoma	3	2.56
Hamartoma not specified	32	27.35
Angiomyolipomatous	2	1.71
Myxoid calcified	1	0.85
Intramuscular capillary	1	0.85
With ectopic meningothelial elements	2	1.71
Vallecula	2	1.71
Salivary glands	1	0.85
Palatine tonsil	2	1.71
Odontogenic epithelium	3	2.56
Fibrous	2	1.71
Fibrovascular	2	1.71
Leiomyomatous	29	24.79
Eruption mesenchymal calcified	2	1.71
Mesenchymal	2	1.71
Myoepithelial	1	0.85
Glial mixed	1	0.85
Neuroepithelial	5	4.27
Neuromuscular/benign triton tumor	3	2.56
Odontogenic	2	1.71
Calcified odontogenic	1	0.85
Inflamed odontogenic	1	0.85
Rhabdomyomatous	5	4.27
Vascular	3	2.56
Peripheral compound odontoma	8	6.84
Hamartomatous sialolipoma	1	0.85
Total	117	100

**Table 7:** Histopathological diagnosis of choristomas reported in the literature.

Types of choristomas	Number of cases	%
Choristoma not specified	3	1.36
Cartilaginous/chondroid	24	10.91
Cerebral/glial/neural/neuroglial/glioma	28	12.73
Cystic	54	24.55
Gastric mucosal	14	6.36
Salivary glands	8	3.64
Epidermal	3	1.36
Respiratory and gastric epithelium	1	0.45
Follicular	2	0.91
Gastrointestinal	3	1.36
Gastrointestinal and pancreatic	2	0.91
Odontogenic	3	1.36
Osseous	40	18.18
Osteochondromatous	1	0.45
Tonsillar	3	1.36
Ectopic thyroid	31	14.09
Total	220	100

## Data Availability

The data supporting this study's findings are available from the corresponding author upon reasonable request.
